# Draft Genome Sequences of Two Edwardsiella piscicida Strains, JF1307 and JF1411, Isolated from Diseased Olive Flounder (Paralichthys olivaceus) Cultured in Japan

**DOI:** 10.1128/genomeA.00600-18

**Published:** 2018-06-28

**Authors:** Hidehiro Sugiura, Shinya Monno, Hazuki Yamashita, Yusuke Kato, Masayuki Imajoh

**Affiliations:** a Department of Bioresource Production Science, The United Graduate School of Agricultural Sciences, Ehime University, Matsuyama, Ehime, Japan; b Laboratory of Fish Disease, Faculty of Agriculture, Kochi University, Nankoku, Kochi, Japan

## Abstract

Edwardsiella piscicida strains JF1307 and JF1411 were isolated from cultured olive flounder that were diagnosed as being infected with edwardsiellosis. The draft genome sequences of the two isolates comprise 3,882,000 bp and 3,827,424 bp with G+C contents of 59.5% and 59.6%, respectively.

## GENOME ANNOUNCEMENT

The genus Edwardsiella belongs to the family Enterobacteriaceae and, prior to 2013, consisted of three taxa, E. tarda, E. hoshinae, and E. ictaluri (1). A novel species, E. piscicida (1), was recently described within the group of organisms traditionally classified as E. tarda. In Japan, E. tarda is thought to be the causative agent of edwardsiellosis in economically important cultured aquatic species, such as Japanese eel (Anguilla japonica), olive flounder (Paralichthys olivaceus), and red sea bream (Pagrus major) (2). E. tarda isolates from red sea bream are also designated atypical because their phenotypic characteristics differ from those of E. tarda isolates from Japanese eel and olive flounder (3). We isolated two E. tarda-like strains in 2013, strain JF1305, from a diseased olive flounder, and strain RSB1309, from a diseased red sea bream. These were identified as E. piscicida and E. piscicida-like strains, respectively, based on PCR and draft genome sequence data (4). Subsequently, Shao et al. (5) reclassified strain RSB1309 as the novel species E. anguillarum. Since both of these novel Edwardsiella species were isolated from diseased fish, we believe that they are potential pathogens of aquatic species cultured in Japan.

Here, we report the draft genome sequences of two E. piscicida strains, JF1307 and JF1411, isolated in July 2013 and November 2014 from diseased olive flounder weighing 154 g and 190 g, respectively. The two strains were cultured overnight in 10 ml brain heart infusion broth at 27°C. Genomic DNA was extracted using a Qiagen Genomic-tip 500/G kit and a genomic DNA buffer set. Genome sequencing was performed on a MiniSeq platform (Illumina K.K., Tokyo, Japan), which generated 4,535,357 reads for strain JF1307 and 4,986,379 reads for strain JF1411. The sequencing reads were assembled using CLC Genomics Workbench version 10.1.1 (Qiagen, Tokyo, Japan). The assembly of strain JF1307 consisted of 97 contigs totaling 3,882,000 bp with a G+C content of 59.5%. The assembly of strain JF1411 consisted of 74 contigs totaling 3,827,424 bp with a G+C content of 59.6%. These draft genome sequences were annotated using the Microbial Genome Annotation Pipeline version 2.23 (http://www.migap.org), yielding 3,555 protein-coding sequences (CDSs), 55 tRNAs, and 4 rRNA operons for strain JF1307 and 3,475 CDSs, 50 tRNAs, and 3 rRNA operons for strain JF1411.

Carbohydrate utilization tests using API 50 CH test kits (Sysmex-bioMérieux, Tokyo, Japan) showed that strain RSB1309 utilized l-arabinose and d-mannitol, but strains JF1305, JF1307, and JF1411 failed to utilize these two carbohydrate sources (data not shown). *In silico* analysis revealed that both l-arabinose and d-mannitol utilization operons are absent in the three E. piscicida genomes. These findings will contribute to a deeper understanding of the difference in carbohydrate utilization between E. piscicida and E. anguillarum.

### Accession number(s).

These whole-genome shotgun projects have been deposited in GenBank under the accession numbers BGMJ00000000 for strain JF1307 and BGMK00000000 for strain JF1411. The versions described in this paper are the first versions, BGMJ01000000 and BGMK01000000, respectively.
